# Tau destabilization in a familial deletion mutant K280 accelerates its fibrillization and enhances the seeding effect

**DOI:** 10.1016/j.jbc.2025.108184

**Published:** 2025-01-13

**Authors:** Gary Jen-Wei Chen, Ming-Yun Chang, Xin-Peng Lin, Debapriya Kundu, Yu-Jen Chang, Yun-Ru Chen

**Affiliations:** 1 Genomics Research Center, Academia Sinica, Taipei, Taiwan; 2 Taiwan International Graduate Program in Interdisciplinary Neuroscience, National Yang Ming Chiao Tung University and Academia Sinica, Taipei, Taiwan; 3 Department of Biochemical Science and Technology, National Taiwan University, Taipei, Taiwan; 4 Chemical Biology and Molecular Biophysics Program, Taiwan International Graduate Program, Institute of Biological Chemistry, Academia Sinica, Taipei, Taiwan; 5 Institute of Biochemical Sciences, National Taiwan University, Taipei, Taiwan; 6 Taiwan International Graduate Program in Interdisciplinary Neuroscience, National Taiwan University and Academia Sinica, Taipei, Taiwan

**Keywords:** tau, stability, fibrillization, seeding, tauopathy

## Abstract

Tauopathies cover a range of neurodegenerative diseases in which natively unfolded tau protein aggregates and spreads in the brain during disease progression. To gain insights into the mechanism of tau structure and spreading, here, we examined the biochemical and cellular properties of human full-length wild-type and familial mutant tau, ΔK280, with a deletion at lysine 280. Our results showed that both wild-type and mutant tau are predominantly monomeric by analytical ultracentrifugation. The mutant tau may lose intramolecular contacts and is significantly destabilized assessed by cross-linking mass spectrometry and urea denaturation. Moreover, the mutant tau displayed accelerated fibril formation compared to the wild-type tau. Upon cross-seeding, the wild-type tau was seeded more easily by wild-type seeds than mutant seeds showing that homotypic seeding is more efficient. The wild-type tau was successfully converted to fibrils with mutant signatures by mutant seeds. Live cell cross-correlation fluorescence spectroscopy studies indicated that wild-type tau forms trimeric species and the mutant tau forms a larger assembly and processes higher cell-to-cell transmission. Overall, these findings shed light on the fundamental mechanisms of tau structure/stability, aggregation, and seeding to facilitate future therapeutic development for tauopathies.

Tauopathies cover a range of neurodegenerative diseases characterized by the presence of neuronal tau inclusions, including Alzheimer’s disease (AD), Pick’s disease, progressive supranuclear palsy, corticobasal degeneration, and frontotemporal dementia and Parkinsonism linked to chromosome 17 (1). Under physiological conditions, tau functions as a microtubule-binding protein that stabilizes microtubules. In the disease state, it is hyperphosphorylated and aggregates into neurofibrillary tangles (NFTs), one of the pathological hallmarks of AD, aside from senile plaques comprising amyloid-β (Aβ). NFTs found in the brains of patients with AD are referred to as paired helical filaments (PHFs) or straight filaments (SFs) (2). Tau pathology is correlated better with the dementia stages of patients with AD (3).

Tau is a natively unfolded protein comprising six isoforms because of alternative splicing (4). The tau isoforms ranging from 352 to 441 residues are composed with or without inserts of 29 or 58 residues (1N or 2N) in the N-terminus and with the presence of three or four microtubule-binding repeats (3R or 4R) in the C-terminal half. Two proline-rich domains are present between N domains and repeat domains. The illustration of protein domains of tau is shown in Figure 1*A*. The abnormal assembly of tau into filaments leads to tauopathies. Recently, cryo-electron microscopy of PHFs and SFs isolated from the brains of patients with AD share a common filament core constructed by two protofilaments with residues 306 to 378 forming a cross-β/β-helix structure but are ultra-structurally polymorphic (5). The cross-β structure of fibrils formed by recombinant full-length 2N4R tau has been examined by X-ray diffraction (6). The fibril core is surrounded by a fuzzy coat formed by the N-terminal and C-terminal regions (7).

**Figure 1:**
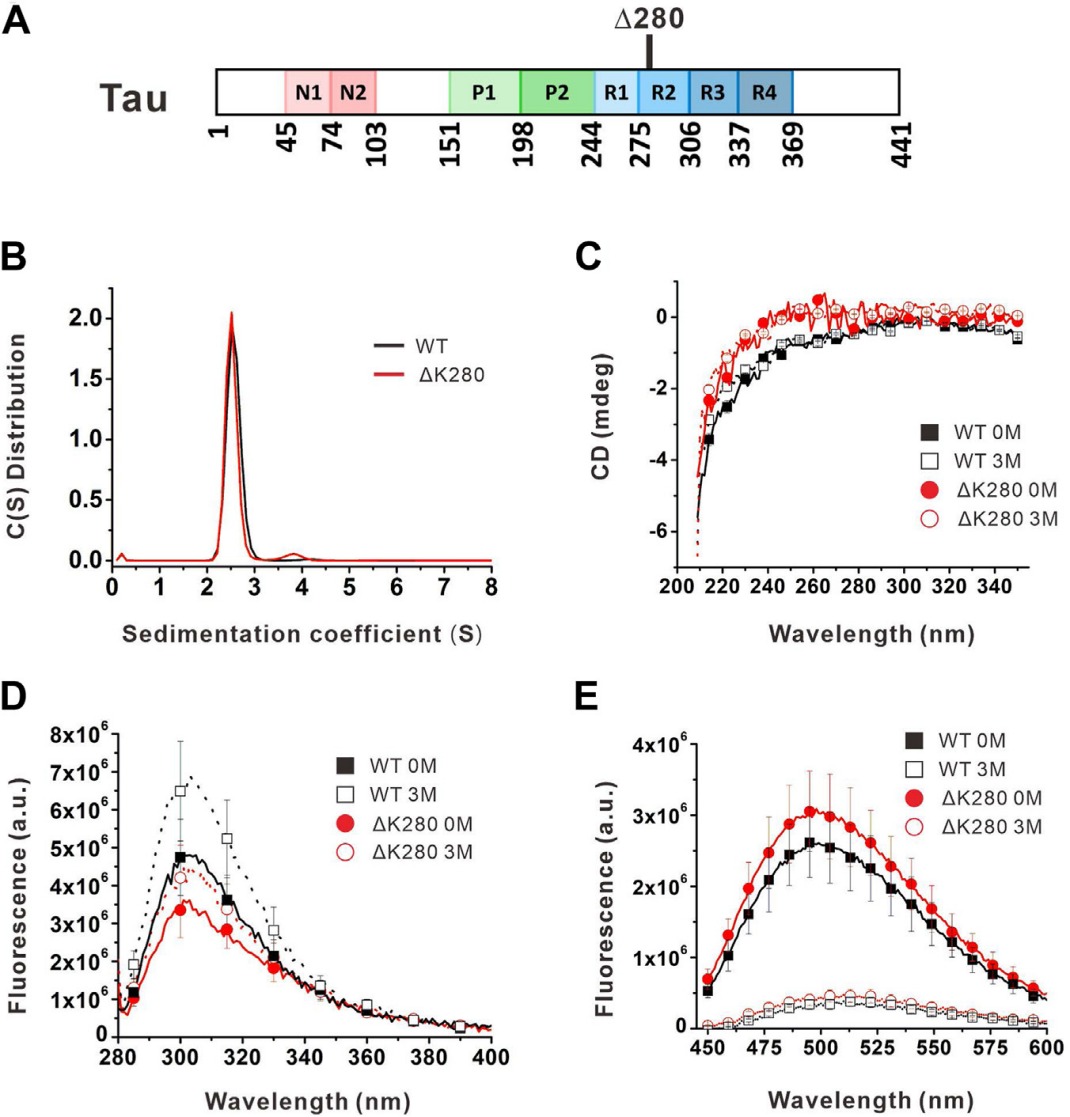
**Assembly and spectral analysis of WT and mutant Tau.***A*, illustraition of Tau protein. *B*, C(s) distribution of tau from SV experiments of AUC. (*C*), Far-UV CD spectra, (*D*) Intrinsic, and (*E*) Bis-ANS fluorescence spectra of tau in native buffer and 3 M GdnHCl containing buffer at 25 ^o^C.

Tau aggregation can be examined *in vitro* in thioflavin T (ThT) assay. ThT is a classic dye for amyloid fibrillization that is chelated onto amyloid fibrils and emits strong fluorescence. Tau aggregation is promoted by negatively charged cofactors such as heparin. Two hexapeptide segments in tau protein, _275_VQIINK_280_ in the second microtubule binding repeat (R2) and _306_VQIVYK_311_ in the third microtubule-binding repeat (R3) are essential for tau aggregation (8). Monomeric tau protein possesses predominantly a random coil structure (9), but some studies showed the existence of residual structures (10, 11). In cellular studies, misfolded tau forms cytoplasmic inclusions, and the extracellular seeds can transfer between cells (12).

Previous studies showed that a tau mutation-bearing deletion at residue K280 (tau ΔK280), which was found in patients with AD and frontotemporal dementia (13, 14), features a higher tendency for aggregation (15). In the present study, we characterized for the first time the folding properties of WT full-length human tau (WT tau, 441 amino acids) and its ΔK280 mutant. Recombinant WT and ΔK280 mutant tau without additional tags were examined by various biophysical techniques for their local structures, folding stability, assembly, fibrillization, and seeding. Furthermore, tau assembly in live cells, cell-to-cell transmission, and extracellular seeding were examined.

## Results

### Assembly and spectral characterization of WT and ΔK280 tau

To gain insights into the possible assembly and structure of tau variants, we purified the longest isoform (tau 441) of recombinant full-length human tau and its K280 deletion mutant (ΔK280) and subjected them to analytical ultracentrifugation (AUC) for species analysis at 5 μM. The tau proteins were subjected to a sedimentation velocity (SV) experiment in AUC, which is a sedimentation method to measure the molecule transportation in solution under centrifugal force and to define the size, shape, and interactions of macromolecules based on hydrodynamic theory. The study provides information on protein species distribution in solution without matrix effect. The results showed that the signals of WT tau and ΔK280 mutant were predominantly situated at ∼2.56 and ∼2.53 S, representing ∼44.4 and ∼45.9 kDa, respectively, which is consistent with the molecular mass of tau monomer 45,850 Da for WT tau and 45,704 Da for ΔK280 mutant. Interestingly, ΔK280 mutant but not WT tau, had a minor peak (∼4%) detected at ∼3.882 S, corresponding to ∼84.9 kDa, indicating that a possible minor dimeric species existed in the mutant form (Fig. 1*B*). No other oligomeric peaks were detected. Our SV result demonstrated that freshly prepared WT and mutant tau are predominantly monomeric.

Although tau is considered intrinsically disordered, residual structure may exist in tau monomer. Hence, we first subjected WT and ΔK280 tau at 2 μM in native buffer or 3 M GdnHCl containing buffer to CD spectroscopy in the far-UV and near-UV regions to examine the secondary and tertiary structures, respectively (Fig. 1*C*). The near-UV CD spectra ranging from 350 nm to 250 nm showed no significant spectral signal and no difference between WT and mutant tau in native and denatured states. The far-UV CD spectra, from 250 nm and below, of both WT and ΔK280 tau showed random coil spectra, indicating that the proteins are intrinsically disordered. Slightly more CD signals were observed in mutant tau than in WT tau. We further examined their intrinsic fluorescence spectra (Fig. 1*D*) by exciting 1 μM tau at 270 nm because tau contains only tyrosine but no tryptophan and monitored the emission from 280 to 400 nm. We found that WT tau possesses higher fluorescence emission than mutant tau in both native and denatured conditions. After denaturation, the intrinsic fluorescence of both WT and ΔK280 tau increased by approximately 40%, which may be attributed to exposure to the residual protein structure. To further examine the possible residual structure of tau, we monitored the protein conformational changes by using an extrinsic fluorescence probe Bis-ANS. Previously, Bis-ANS fluorescence was employed to study the conformational changes of Aβ species and the stability was determined (16, 17, 18). Here, the Bis-ANS spectra showed that native tau has ∼6-fold more fluorescence intensity than denatured tau (Fig. 1*E*). In native conditions, WT tau has slightly lower Bis-ANS fluorescence than mutant tau, but the fluorescence of both is greatly reduced in GdnHCl-induced denaturation. The maximum wavelength of both WT and mutant tau also red-shifted from 500 nm to 512 nm upon denaturation. The result demonstrated that native tau contains residual structures and can be examined by Bis-ANS fluorescence.

### Mutant tau losses intramolecular contacts linked to R2 domain

**Figure 2:**
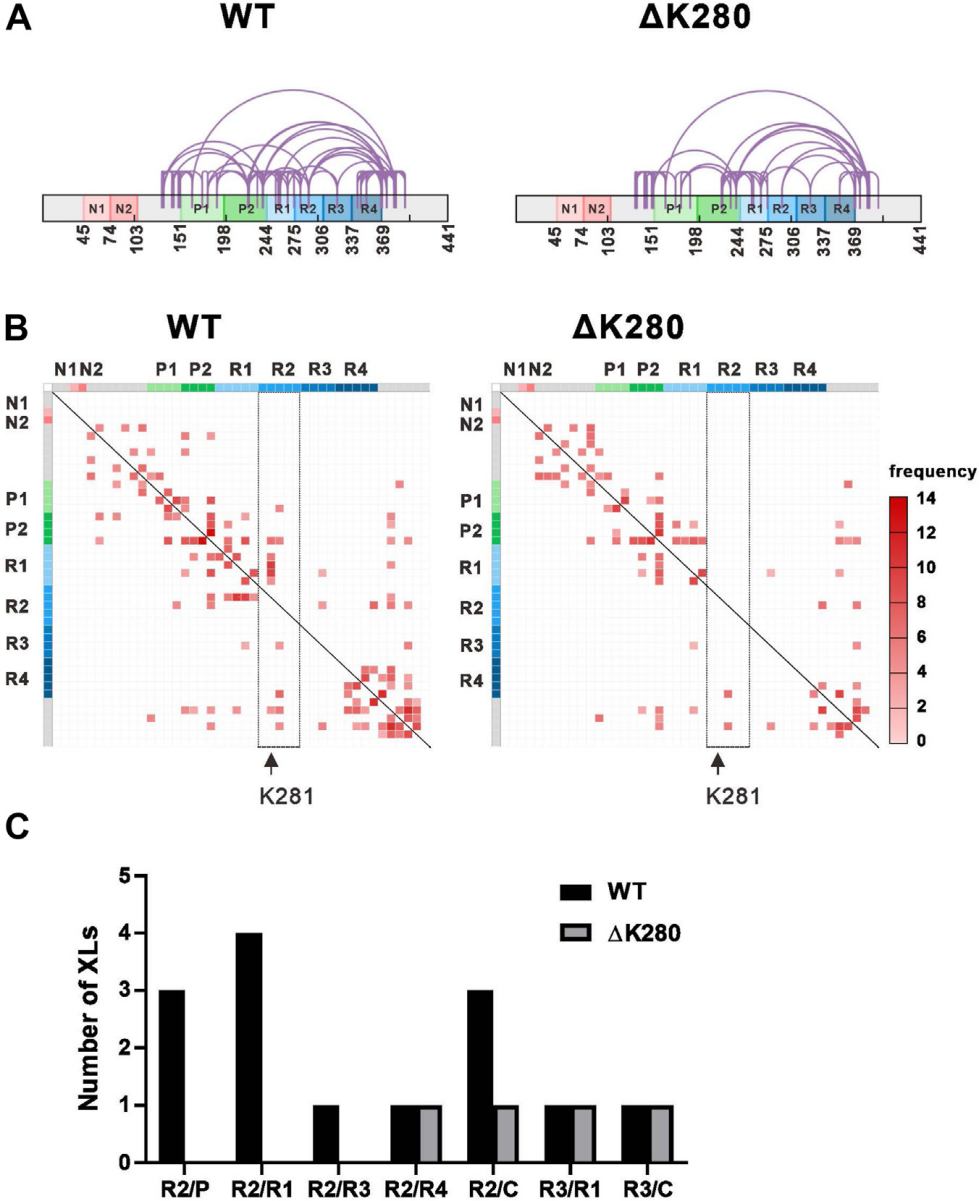
**Consensus cross-links of full-length WT and mutant tau.***A*, schematic drawing of the intramolecular cross-links. The domains of tau are colored. The crosslinks are drawn in purple lines. *B*, heat maps of the consensus cross-links in tau colored by the average frequency of four replicates. The lysine residues are drawn in X and Y axes and the domains are colored accordingly. The cross-links related to residues in the R2 domain are shown in *black* boxes. Residue K281 is indicated. *C*, the number of consensus cross-links in each domain interacting with the R2 or R3 domains.

To determine structural differences residing in wild-type and ΔK280, we examined tau monomer structure with cross-linking mass spectrometry (XL-MS). This study provides structural information derived from distance restraints. The freshly prepared protein was centrifuged and the supernatant was collected for XL-MS. We used a primary amide crosslinker, DSS, to crosslink the lysine residues in proximity. The protein was trypsin digested and subjected to mass spectrometry. The crosslinked contacts detected are illustrated in Figure 2*A*. We observed that cross-link patterns are different in the location of the lysine pairs between WT and ΔK280 tau. The heat maps of cross-linking indicated that in ΔK280 tau the interactions especially linked to the R2 domain were absent (R2/proline-rich, R2/R1, and R2/R3 domains) or reduced (R2 and C-terminus from residues 369–441) (Fig. 2, *B* and *C*), whereas, the number of cross-links remained the same in the contacts between R2 and R4, R3 and R1, and R3 and C-terminus. Compared to other residues in the R2 domain, most interactions connecting residue K281 in the R2 domain with other residues in ΔK280 were lost (K281 paired with K240, K257, K259, K267, and K274). Since our AUC SV results showed that both WT and mutant tau are predominantly monomeric, we suggested that lack of K280 residue primarily contributes to the reduction of intramolecular contacts leading to a conformational change of tau especially in the R2 domain. However, we cannot exclude the possibility that there are sparse oligomers present and some of the reduction may be from intermolecular contacts.

### Equilibrium folding study showed that tau adopts a 2-state equilibrium folding mechanism where tau mutant is destabilized

**Figure 3:**
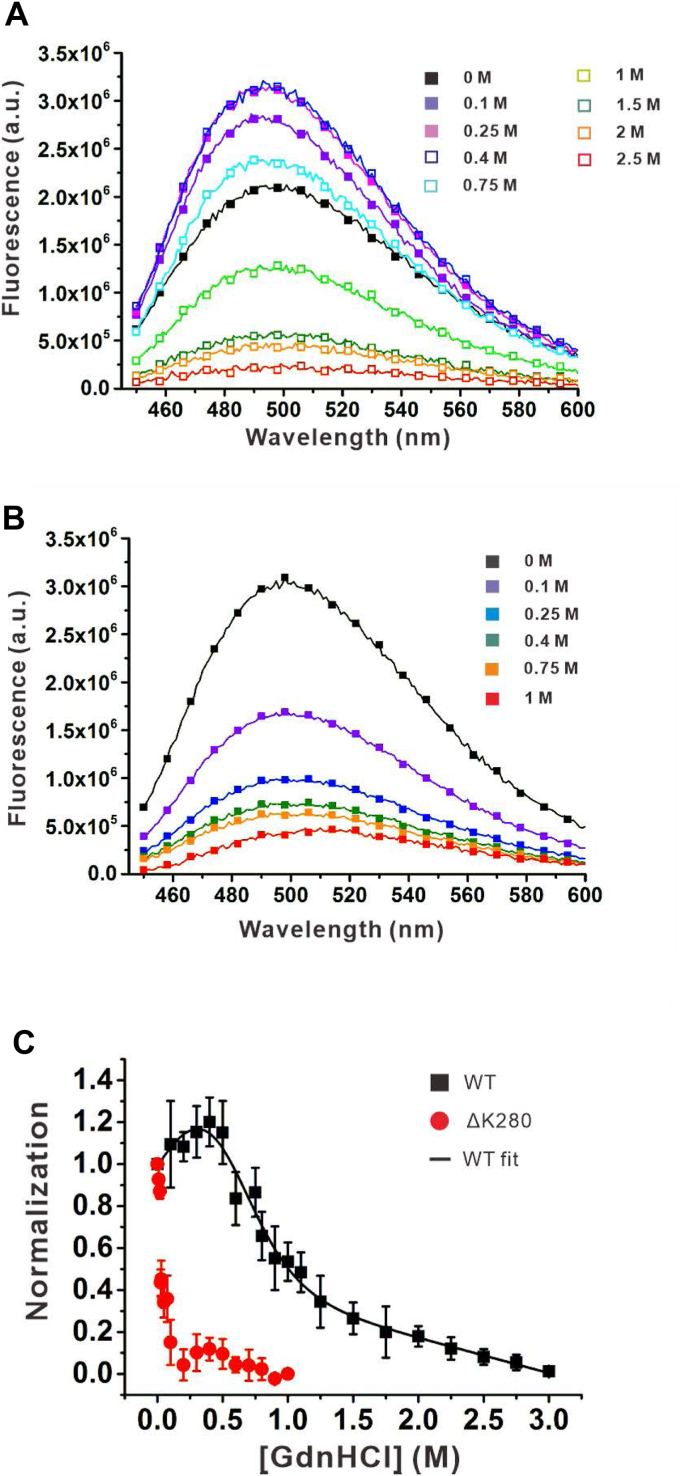
**Folding stability of tau from GdnHCl denaturation.** Bis-ANS fluorescence spectra of (A) WT and (B) ΔK280 tau at 1 μM were monitored in various GdnHCl concentrations at 25 °C. *C*, GdnHCl denaturation of tau. Bis-ANS emission of tau at 500 nm was averaged, normalized to the signal in native condition, and plotted against GdnHCl concentration. The data were fitted to a two-state folding mechanism. The fit is shown in a solid line.

Considering that native and denatured tau significantly differ in Bis-ANS fluorescence, we performed an equilibrium folding study by Bis-ANS fluorescence of tau in GdnHCl ranging from 0 to 3 M. The WT and mutant tau at 1 μM were equilibrated with different concentrations of GdnHCl in Tris buffer and subjected to Bis-ANS fluorescence measurement. Our results showed that the Bis-ANS fluorescence intensity of WT tau slightly increased then decreased (Fig. 3*A*), whereas the Bis-ANS fluorescence of mutant tau decreased while increasing GdnHCl concentration. (Fig. 3*B*). The intensity indicates the conformational changes of the solvent-exposed hydrophobic cluster on the surface of the tau that Bis-ANS binds. The fluorescence intensity in different GdnHCl concentrations was collected, normalized to the native signal, and then plotted against GdnHCl concentration (Fig. 3*C*). We found the denaturation curve of WT tau adopted a two-state equilibrium folding mechanism. The pre-transition is from 0 to 0.3 M GdnHCl followed by a transition from 0.3 M to 1.25 M and a post-transition state after 1.25 M. The Gibbs free energy of folding, ΔG_UN_, obtained from the fit is 1.55  ± 0.74 kcal/mol, and the m-value is 2.95  ± 0.73 kcal/mol/M. The calculated midpoint [GdnHCl]half is ∼0.53 M GdnHCl. By contrast, the mutant tau ΔK280 showed no pre-transition but an immediate decline in the Bis-ANS signal upon denaturation. The transition is from 0 to 0.25 M GdnHCl. The mid-point of mutant tau is around 0.025 M GdnHCl. The result demonstrated that WT tau adopts a two-state equilibrium folding model and that ΔK280 tau is much less stable than WT tau.

### ΔK280 tau fibrillizes faster than WT tau and their aggregates adopt distinct molecular structures

**Figure 4:**
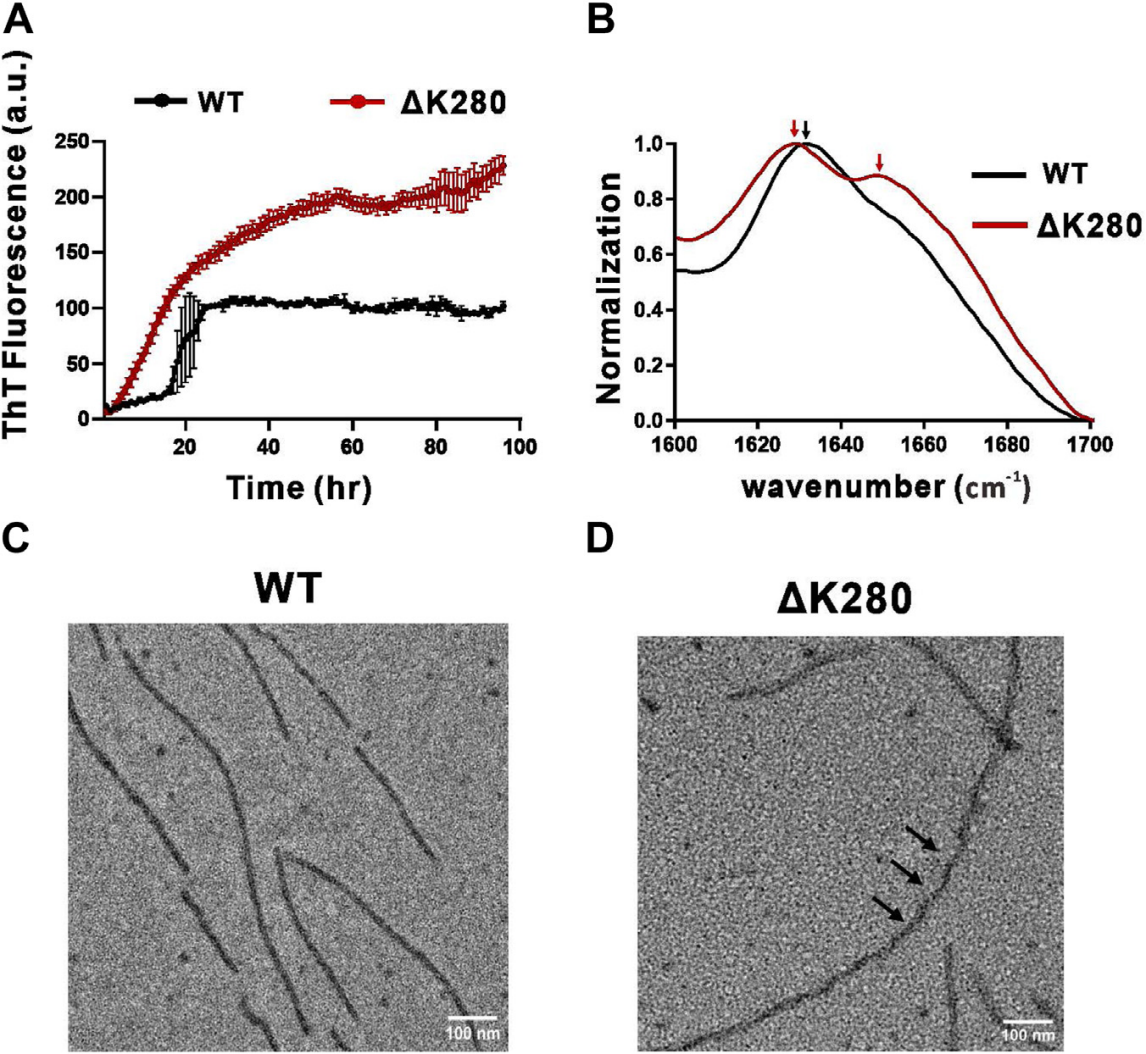
**Fibrillization of WT and ΔK280 tau.***A*, Fibrillization assay of WT and ΔK280 tau monomers induced by heparin. ThT assay was performed. *B*, FTIR spectra of WT and mutant tau fibrils. FTIR data between 1600 and 1700 cm^−1^ were collected and normalized. *C* and *D*, TEM images of WT and mutant tau fibrils. *Black* triangles indicate periodic twists observed in ΔK280 fibrils. Scale bar, 100 nm.

To study the differences between WT and ΔK280 mutant tau aggregation, we used heparin as a cofactor to induce tau aggregation and monitor fibril growth by ThT fluorescence for ∼100 h at 25 °C. The results showed that both WT and ΔK280 mutant tau fibrillized, whereas ΔK280 mutant fibrillized faster than WT tau. The mutant tau showed a rapid elongation phase without a lag time, whereas WT tau followed the classic fibrillization with a lag time of ∼18 h, followed by an elongation phase and saturation phase (Fig. 4*A*). The ThT intensity of WT tau at the end stage was lower than that of the mutant tau. To examine the molecular structure of the fibrils, the final products from the aggregation experiment were centrifuged, resuspended in deuterated water, and then subjected to FTIR to study the secondary structure of the aggregates. The amide I region in FTIR from 1600 cm^−1^ to 1700 cm^−1^ were compared. The WT aggregates showed a major peak at 1632 cm^−1^ indicating β-sheet structure (19) and a small shoulder at 1653 cm^−1^, whereas ΔK280 aggregates showed a strong peak at 1629 cm^−1^ which is classic for β-sheet structure (20), and a prominent shoulder at 1649 cm^−1^ for alpha-helix (Fig. 4*B*). To examine the morphology, we subjected the end-point products to TEM. We found that WT tau formed abundant mature fibrils that are elongated and straight (Fig. 4*C*). By contrast, ΔK280 mutant under the same condition formed fibrils with periodic twists observed by the twisted fibril morphology with a thinner width (Fig. 4*D* and Fig. S1, indicated by arrowheads). It is estimated that one out of every two to three fibrils had such twists. The result demonstrated that ΔK280 mutant forms fibrils much faster and the fibrils have distinct molecular signatures to WT fibrils.

### ΔK280 mutant possesses a higher propensity for **β**-sheet formation and its seeds demonstrate superior seeding ability

**Figure 5:**
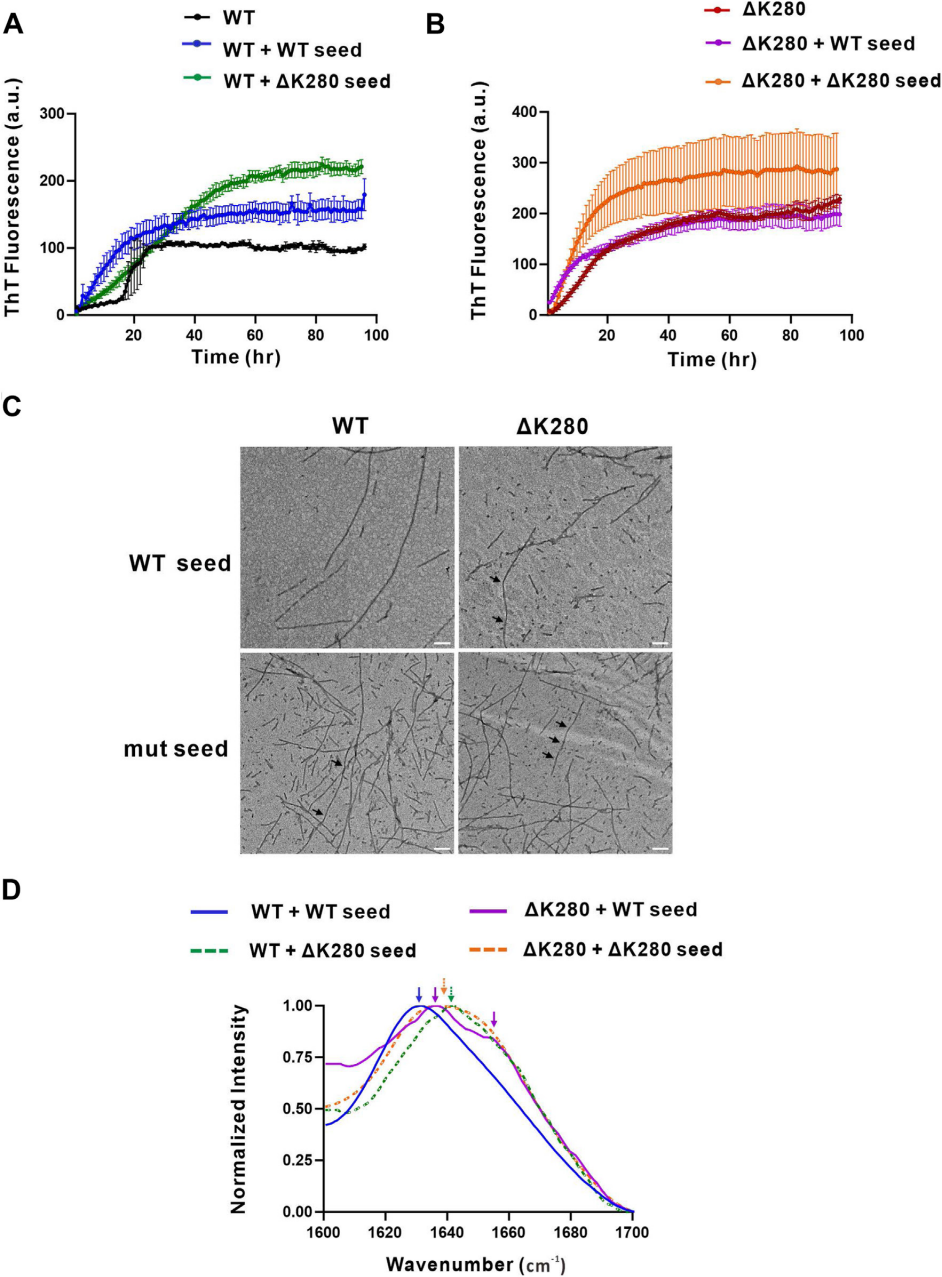
**Seeding assay of WT and ΔK280 tau induced by preformed fibril seeds.***A*, WT or (B) mutant tau monomers in the presence or absence of preformed fibril seeds, incubated at 25 ^o^C in the presence of ThT and heparin. Signals were subtracted from the buffer background. *C*, TEM images of the end-point products from tau seeding assay. Scale bar, 100 nm. *D*, FTIR spectra of end products of the seeding assay. Data between 1600 and 1700 cm^−1^ were collected, normalized, and shown.

To investigate seeding abilities, we first collected WT and mutant Tau fibrils seeds after sonication of the preformed fibrils. These seeds were then introduced to recombinant WT (Fig. 5*A*) or mutant tau monomer (Fig. 5*B*) in a 5% v/v ratio to induce fibril growth. Thioflavin T fluorescence assay was used to monitor fibrillization as described above. In the study to seed WT tau monomer, we found that WT seeds were able to seed WT tau monomer effectively, compared to the one without seeds (Fig. 5*A*), as evidenced by the disappearance of the lag phase. The mutant seeds showed less seeding effect to WT tau monomer compared to the WT seeds although the final ThT intensity with the mutant seed was higher. In the case of mutant tau monomer, the addition of WT tau seeds showed a seeding effect that accelerated the elongation phase, while the addition of mutant tau seeds showed a greater seeding effect resulting in increasing faster elongation and higher final ThT intensity. The differences observed in the seeding effect indicate the existence of a species barrier between WT and mutant tau (Fig. 5*B*).

To further understand the molecular structure of seed-induced fibrils, we conducted TEM imaging (Fig. 5*C*) and FTIR experiments (Fig. 5*D*) on the end-point products with and without seeding. We found that WT seeds’ addition to WT monomer resulted in long extended fibrils similar to WT monomer, with a peak at 1632 cm^−1^ representative of β-sheet structure. However, the addition of ΔK280 seeds to the WT monomer resulted in more intense fibrilization. The fibril morphology became more mutant-like as evidenced by twists observed in TEM and a peak at ∼ 1642 cm^−1^ measured in FITR. When the addition of WT seeds to ΔK280 monomer was done, the mutant tau fibrils adopted more mixed morphology with both straight and twisted fibrils, where we observed twists in one out of every 5 to 6 fibrils, as shown in Figure S2*A*. The FTIR spectra showed a prominent peak at 1636 cm^−1^ for β-sheet and shoulder at 1654 cm^−1^ for α-helix respectively. The minor shoulder at ∼1654 cm^−1^, a characteristic of α-helix, suggests the coexistence of β-sheet and α-helix structures which has been implicated in isolated human tau fibrils and PHFs from AD patients (19). While the addition of ΔK280 fibril seeds to ΔK280 monomer, the mutant fibrils had periodic twists as shown in Figure S2*B*. There were apparently more twists in comparison to the WT seeds addition, where 2 or 3 fibrils out of every 5 to 6 fibrils had twists. The FTIR spectra showed a broad peak at ∼1637 to 1651 cm^−1^. The results demonstrate that a difference existed in seeding properties between WT and ΔK280 Tau seeds to WT monomer, where the latter alters WT to fibrillize into ΔK280-like species. The seeding effect of WT seeds to ΔK280 monomer was less efficient, maybe due to the fast self-aggregating nature of ΔK280 monomer. The FTIR profile is summarized in Table 1. The enhanced β-sheet characteristics in FTIR spectra of fibrils containing mutant tau are consistent with the increased ThT signal observed in fibrilization and seeding assay.

**Table 1:** FTIR peak positions of tau fibrils

Tau samples	**α**-helix (cm^−1^)	**β**-sheet (cm^−1^)
WT		∼1632
ΔK280	∼1649	∼1629
WT + WT seed		∼1632
WT + mut seed	∼1642	
ΔK280 + WT seed	∼1654	∼1636
ΔK280 + mut seed	∼ 1651	∼1637

mut, ΔK280 mutant.

### Tau ΔK280 mutant forms larger intracellular species in live cell

**Figure 6:**
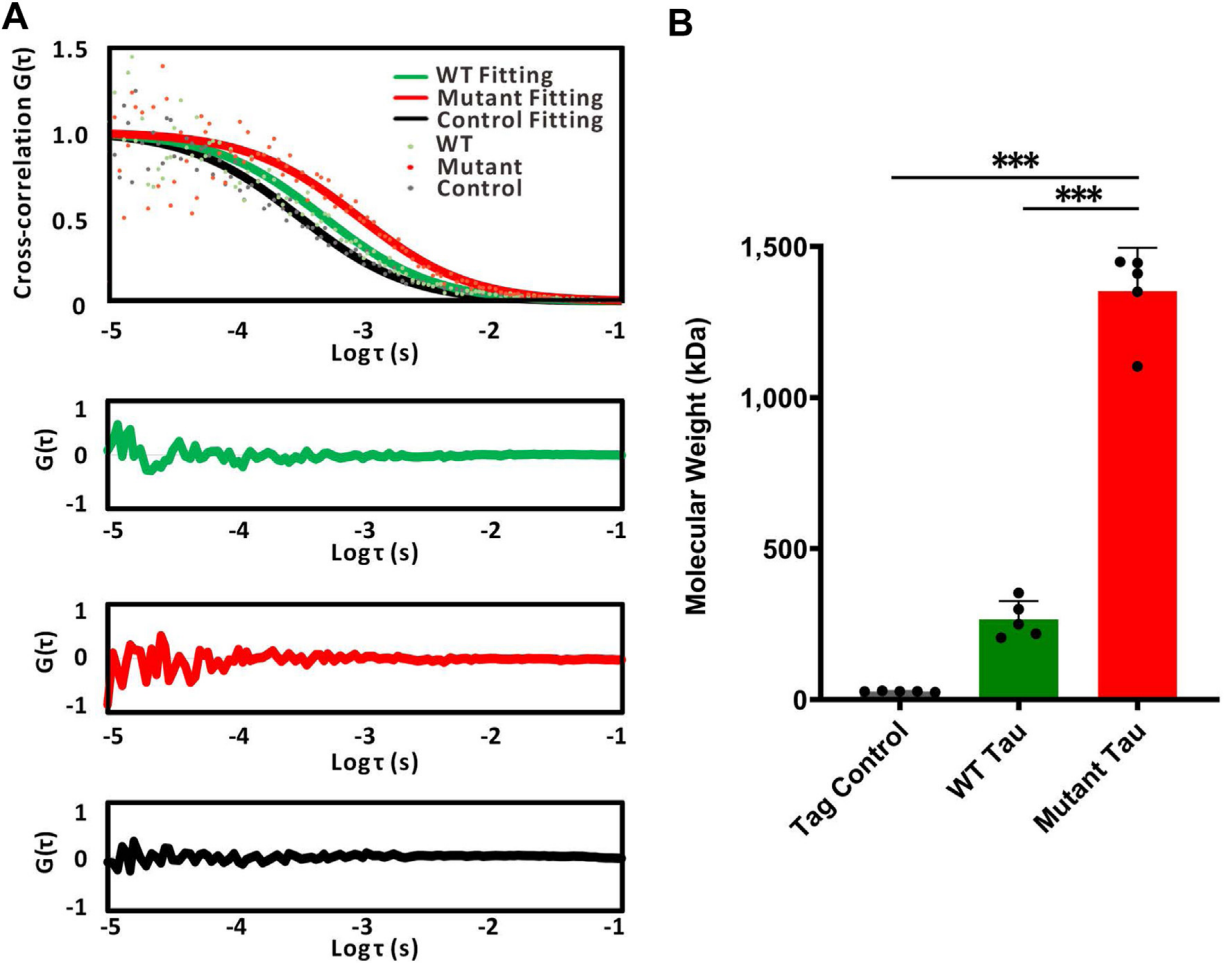
**Intracellular WT and ΔK280 tau species detected by FCCS in live cells.***A*, Cross-correlation of intracellular tau. The cross-correlation data and their fitting curves of WT tau (*green*), ΔK280 mutant tau (*red*), and the fusion control (*black*) are shown. The corresponding fitting residuals are shown below. *B*, the calculated molecular weight of WT and ΔK280 tau. Results are mean ± s.e.m. of five independent experiments. The statistical analysis was performed by one-way ANOVA with Tukey’s multiple comparison post-test (∗, *p* < 0.05; ∗∗, *p* < 0.01; ∗∗∗, *p* < 0.001).

To investigate tau protein behavior in live cells, we utilized Fluorescence Correlation Spectroscopy (FCS) to monitor the Brownian motion of free tau. FCS enables us to analyze the fluorescence fluctuations caused by particle movement, which helps in determining the size of the tau assemblies. We overexpressed N-terminal eGFP-fused wild-type (WT) or ΔK280 mutant tau in HEK293T cells to observe their intracellular behavior. The correlation curve (Fig. 6*A*) and molecular mass derived from the FCS data were used to estimate the size of the tau assemblies (Fig. 6*B*). Since the relative molecular mass is inversely proportional to the cube of diffusion coefficient (21), the assembly of tau is estimated by comparing it to the theoretical molecular mass of eGFP-tau WT (72.79 kDa) and eGFP-tau ΔK280 (72.64 kDa). From the data, we detected a trimeric species in WT tau (D = 36.00 ± 1.97 um^2^/s, 201.40 ± 35.42 kDa), whereas a higher-molecular-weight (HMW) species with a smaller diffusion coefficient (D = 19.00 ± 1.02 um^2^/s, 1,369.12 ± 236.96 kDa) estimated to be around 19-mer (∼16–22 mer) was detected in the mutant tau expressing cells. In this study, we only examined the tau species that were not bound to microtubules (MTs) since the MT-bound tau will lead to very slow mobility as reported in previous literature (22, 23).

### Tau ΔK280 mutant transmits in higher ratio among cells

**Figure 7:**
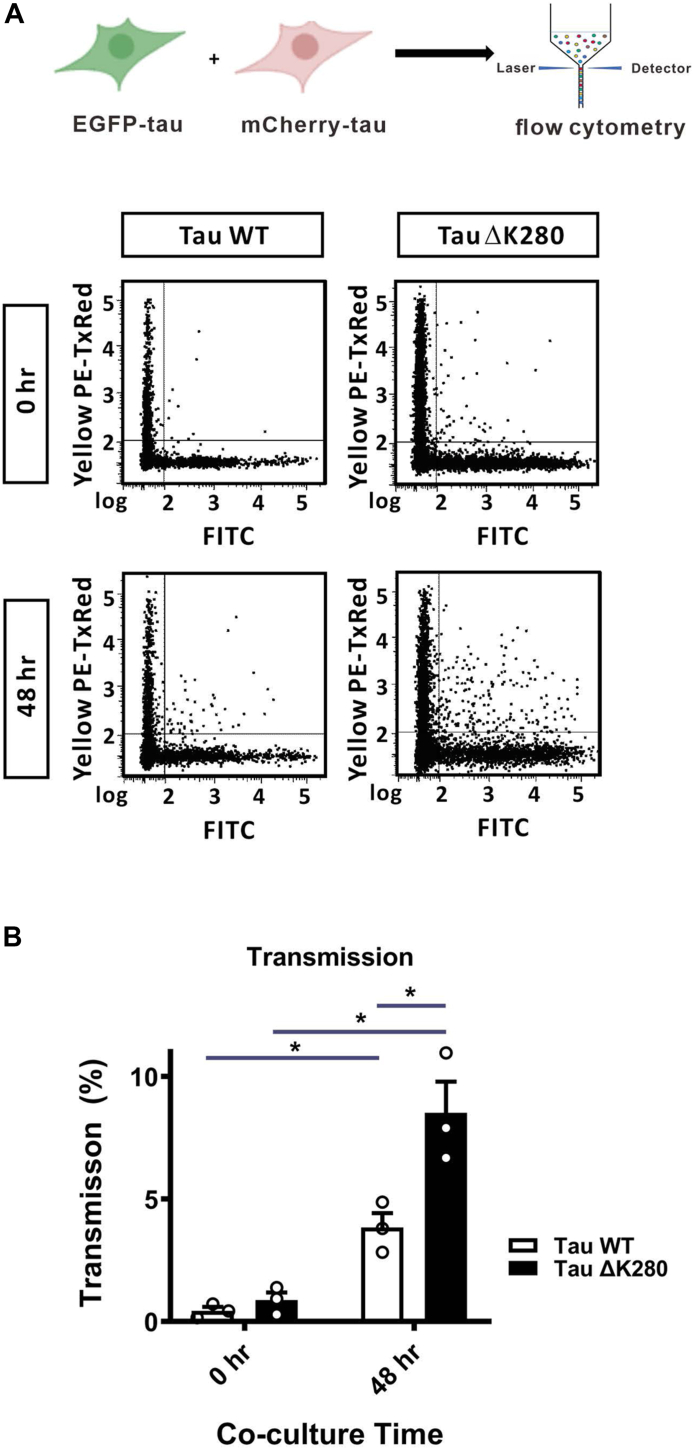
**Transmission of tau examined by flow cytometry.***A*, flow cytometry of co-culture experiments of eGFP-tau WT and mCherry-tau WT as well as eGFP-tau ΔK280 and mCherry-tau ΔK280 at time 0 and 48 h. *B*, quantitative results of transmission by flow cytometry. The statistical analysis was performed by two-way ANOVA with Tukey’s multiple comparison *post hoc* test (∗, *p* < 0.05; ∗∗, *p* < 0.01; ∗∗∗, *p* < 0.001).

We examined the cell-to-cell transmission behavior of WT and mutant tau. Both mCherry- or eGFP- WT and mutant tau constructs were prepared, and a plasmid with mCherry-eGFP tandem protein was used as a duo-fluorescence control. For the cellular transmission of WT tau, we transfected mCherry-WT tau and eGFP-WT tau to HEK293T cells separately overnight and then co-cultured the two populations of cells in equal amounts, *i.e.* 6 × 10^5^ cells/well, to reach 80% confluence. The samples were harvested for subsequent flow cytometry monitoring the green and red fluorescence at 0 and 48 h after co-culturing. Similarly, for the cellular transmission of mutant tau, we transfected mCherry-tau ΔK280 and eGFP-tau ΔK280 to HEK293T cells overnight, then, co-cultured them following the same procedure for WT tau. The cells with dual fluorescence indicated tau transmission among cells. The transmission was indicated by the percentage of cells with dual fluorescence in all fluorescent cells (*i.e.*, single and double fluorescence). Flow cytometry results showed that dual fluorescence increased in the co-cultured cells with eGFP-tau WT and mCherry-tau WT from 0.4 ± 0.3% to 3.8 ± 1.0% from time 0 to 48 h (Fig. 7). In addition, the transmission in eGFP-tau ΔK280 co-cultured with mCherry-tau ΔK280 increased from 0.9% ± 0.6% to 8.5% ± 2.2% from time 0 to 48 h. The transmission population of tau ΔK280 mutant (8.5% ± 2.2%) was 2.23-fold to that of WT tau (3.8% ± 1.0%). The results demonstrated cell-to-cell tau transmission in both WT and ΔK280 mutant, where ΔK280 mutant possesses higher transmission properties.

**Figure 8:**
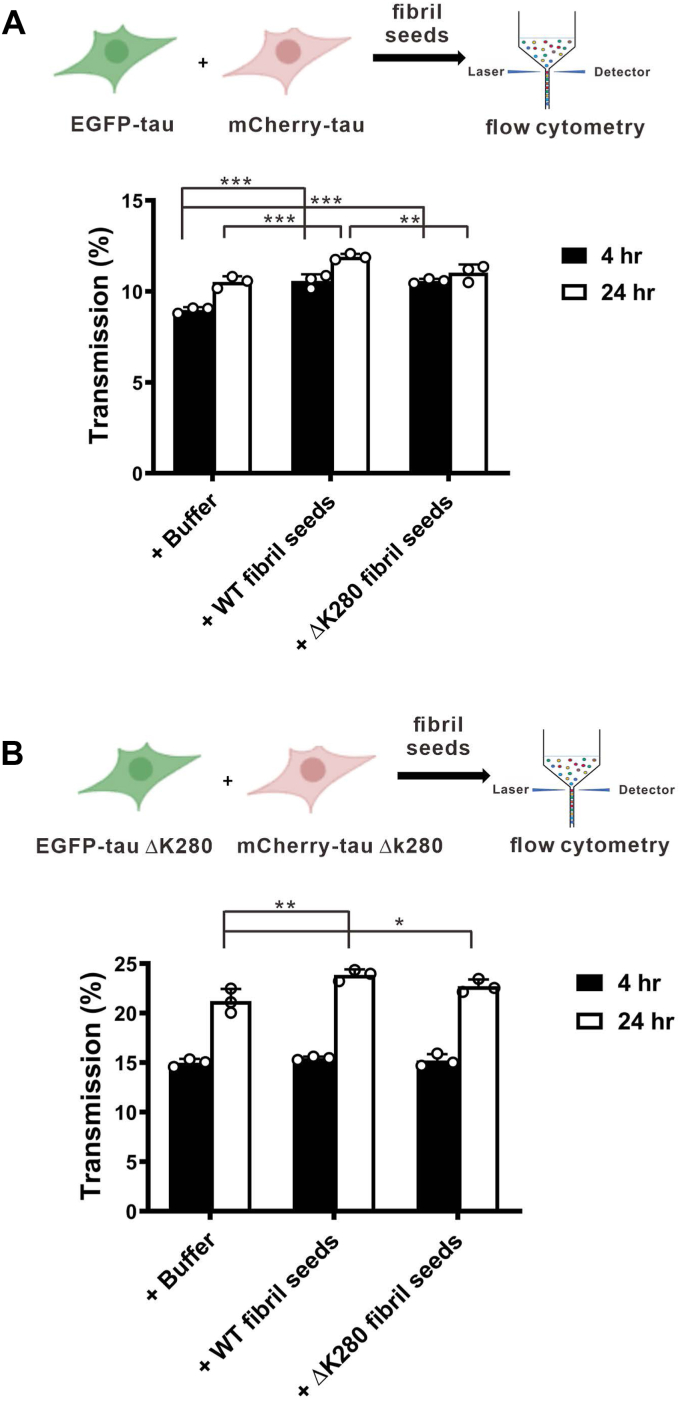
**Transmission of Tau with Tau fibril seeds by flow cytometry.***A*, flow cytometry of co-cultured cells expressing eGFP-tau WT and mCherry-tau WT, which were treated with WT or mutant tau fibril seeds for 4 and 24 h. *B*, flow cytometry of co-cultured cells expressing eGFP-tau ΔK280 and mCherry-tau ΔK280, which were treated with WT or mutant tau fibril seeds for 4 and 24 h. The statistical analysis was performed by two-way ANOVA with Tukey’s test (∗, *p* < 0.05; ∗∗, *p* < 0.01, ∗∗∗, *p* < 0.001).

We further tested the seeding effect by adding WT or mutant fibril seeds to the above experiments. Cells expressing eGFP-WT tau and mCherry-WT tau (Fig. 8*A*) were co-cultured and treated with WT and mutant tau fibril seeds at 5 μM for 4 and 24 h, and then subjected to flow cytometry. The treatment of buffer containing 20 mM Tris-HCl, pH 7.4 was used as a negative seeding control. The transmission percentage was calculated. Similarly, the cells expressing eGFP-tau ΔK280 and mCherry-tau ΔK280 were examined following the above procedure for WT tau (Fig. 8*B*). The results showed that the cells expressing mutant tau had higher transmission, 15% at 4 h and 21% at 24 h, than that of the cells expressing WT tau, 9% at 4 h and 11% at 24 h. This result is consistent with our previous data. Treatment of WT co-culture with WT or mutant fibril seeds showed that the extracellular seeds increased slightly but significantly than the buffer control at 4 h after treatment (Fig. 8*A*). At 24 h, WT fibril seeds, but not the mutant seeds, significantly increased the transmission than the buffer control. This result suggests that the WT seeds retained better seeding properties than WT co-culture, which is consistent with the result from the *in vitro* seeding assay. In the mutant co-culture (Fig. 8*B*), the transmission was not different at 4 h, but increased significantly at 24 h than the buffer control from 21% to 24% and 23% for WT and mutant seeds, respectively. No difference in transmission was observed between WT and mutant seeds. Overall, the results showed that increased transmission upon fibril treatment exhibited an extracellular seeding effect on the intracellular tau. We also examined the seeding effect of monomers in the above cellular transmission study (Fig. S3). Interestingly, at 24 h there was also a significant increase in transmission for WT and ΔK280 monomer seeds after monomer addition in either WT tau expression cells (Fig. S3*A*) or mutant tau expressing cells (Fig. S3*B*). The mutant monomer seeds are more potent than the WT monomer seeds in both cell systems. The result may suggest monomer incubated in the media aggregated into a species that is capable of seeding the intracellular tau where mutant tau is more potent than the WT tau.

## Discussion

Tau is an intrinsically disordered protein; however, how disordered, monomeric tau ultimately aggregates into highly ordered fibril structure is not fully understood. Here, for the first time, we employed a denaturation study for tau stability using Bis-ANS studies. We also employed XL-MS to examine intramolecular contacts in native tau structures. Our results showed a significant decrease in Bis-ANS fluorescence from native to unfolded tau in both WT and mutant, demonstrating the existence of native tau conformation, which is consistent with the previous literature reporting a hairpin-like structure using FRET on various Trp mutant tau (24) and NMR studies showing the existence of residue structures in tau (11). The GdnHCl denaturation study showed that WT tau follows a two-state equilibrium folding model that allows us to calculate the Gibbs free energy of human tau protein. The disease-related ΔK280 mutant with a single deletion in the R2 repeat of the microtubule-binding domain is much destabilized. This result is consistent with our XL-MS data showing the loss of several contacts between R2 and R3 and other domains. Previous studies showed that ΔK280 in K18 increases the hydrophobic surface at the hexapeptide _275_VQIINK_280_ region (15). The NMR study showed that K280 resides in a transient β-structure in residues 274 to 284 (11) comprising an aggregation-prone hexapeptide _275_VQIINK_280_ and also close to the other aggregation-prone hexapeptide _306_VQIVYK_311_ (25). Hence, deletion at K280 may affect K281 interaction with other intramolecular residues resulting in destabilization and further exposure of the fibril core for fibrilization.

The destabilization and lack of intramolecular contacts at the R2 region in the ΔK280 mutant tau enable easier intermolecular interactions, leading to rapid aggregation without a noticeable lag phase. In addition to its accelerated and abundant fibrilization, the mutant also demonstrates a distinct β-sheet propensity in its structural characteristics. Conversely, the structurally stable wild-type tau requires a longer nucleation process before entering the elongation step. In the *in vitro* seeding experiments, the fragmented seeds act as readily available templates to expedite the lag phase. Our result showed that homotypic seeding is more favorable than heterotypic seeding, where WT tau adopts the molecular signature to the WT seeds better than the mutant. This could be due to the fast self-aggregation property of the mutant tau.

In our live cell FCS studies, we found WT tau formed a trimer species, but mutant tau formed larger aggregates, possibly a trimeric assembly to ∼6 fold. Previously, tau trimer has been identified as the minimal unit by characterizing recombinant tau repeat domain and AD brain-derived tau (26, 27). In addition, our co-culture model displayed different transmission properties between WT and ΔK280 mutant, in which the transcellular tau population in ΔK280 mutant was nearly 2-fold greater than that in the WT co-culture group. The result demonstrated that differences reside in the transmission for different tau species and suggests that the cells bearing relatively larger tau oligomers possess a better transmission rate. Although the difference in using WT or mutant fibril seeds in cellular seeding was not obvious, there is still a significant difference in favoring homotypic fibril seeding in the WT tau-expressing cells. However, the same phenomenon was not observed in the monomer seeding study in cells.

Several potential mechanisms may underline the differences observed in the seeding assays *in vitro* and cells. In cells, exogenous tau seeds need to enter the plasma membrane to seed intracellular tau. It was reported that heparan sulfate or specific receptors, such as LRP1, bind and mediate endocytosis of tau (28, 29, 30). The differences in interaction of extracellular tau to heparan sulfate or LRP1 may result in different levels of tau entry leading to subsequent differences in cellular aggregation and transmission. Another possible mechanism lies in the proteostasis systems in cells. In cells expressing WT tau, proteostasis may function effectively in maintaining tau homeostasis. However, mutant tau adopts a pathogenic, aggregation-prone conformation that tends to overwhelm the proteostasis mechanisms. As a result, the cell may expel mutant tau as a means of alleviating this proteotoxic burden (31, 32, 33). In summary, we provide a mechanism for destabilization and structure conversion in seeding reaction after a comprehensive characterization of tau WT and ΔK280 mutant regarding their structure, stability, aggregation, seeding, and transmission. This study serves as an important basis for understanding the physical features residing in physiological and familial mutant tau and facilitates therapeutic development in tauopathies.

## Experimental procedures

### Tau cloning

The plasmid of pRK5-EGFP-tau was purchased from Addgene and tau was then subcloned to pET-29b (+) plasmid (EMD Biosciences) by *Nde*I and *Eco*RI sites. Tau ΔK280 mutant was generated by site-directed mutagenesis. For mammalian expression constructs, WT tau, mutant tau, and mCherry-eGFP tandem protein were cloned into mammalian expressing vector pEGFP-C1 and pmCherry-C1 (Addgene). Tau ΔK280 mutation was also generated by site-directed mutagenesis. The constructs made were enhanced green fluorescent protein-tagged WT htau 441(eGFP-tau WT), eGFP-tagged htau 441 ΔK280 mutant (eGFP-tau ΔK280), mCherry-tagged WT htau 441 (mCherry-tau WT), mCherry-tagged htau 441 ΔK280 mutant (mCherry-tau ΔK280), or, mCherry-eGFP tandem protein for fluorescent duo-positive control. All constructs were confirmed with double sequencing.

### Expression and purification of tau

A single colony of WT or mutant Tau441-pET29b transformed in *E. coli* BL21 (DE3) was incubated in 1 L LB broth with 50 μg/ml kanamycin at 37 °C with shaking at 200 rpm until OD_600_ ∼ 0.8, and then induced by 1 mM IPTG at 37 °C for 3 h. Cells were harvested by centrifugation at 4 °C. After removing the supernatant, the pellet was suspended in 30 ml of lysis buffer containing 20 mM MES, pH 6.5, 1 mM MgCl_2_, 100 mM NaCl, 2 mM EDTA, 5 mM DTT, and a protease inhibitor (Merck). The cells were then lysed by a microfluidizer and boiled at 80 °C for 30 min, followed by centrifugation at 4 °C. The supernatant was collected and purified by FPLC using an ion exchange column (SP Sepharose HP; GE Healthcare Biosciences) with loading buffer (20 mM MES, pH 6.5, 1 mM MgCl_2_, 5 mM NaCl, 2 mM EDTA, and 1 mM DTT) and eluting buffer (20 mM MES, pH 6.5, 1 mM MgCl_2_, 500 mM NaCl, 2mM EDTA, and 1 mM DTT). Fractions containing tau were collected and dialyzed to dialysis buffer (20 mM MES, pH 6.5, 1 mM MgCl_2_, 5 mM NaCl, 2 mM EDTA, and 1 mM DTT). After that, the protein was further purified by FPLC using a Heparin HP column (GE Healthcare Biosciences) with loading buffer and eluting buffer mentioned above. Tau fractions were collected and concentrated by ultrafiltration. After dialysis to 20 mM Tris-HCl, pH 7.4, pure tau protein was stored at −20 °C. For the experiments, tau was centrifuged at 17,000*g* for 20 min at 4 ^o^C and the supernatant was quantified by BCA assay.

### Intrinsic fluorescence and circular dichroism spectroscopy

Fluorescence emission spectra were obtained using a FluoroMax-3 spectrofluorometer (Horiba Jobin Yvon). The spectra were collected from 280 nm to 400 nm with excitation at 270 nm performed at 25 ^o^C maintained by circulating water bath. Far-UV and near-UV circular dichroism (CD) spectra were collected by a Jasco J-815 spectropolarimeter (Jasco) with 1 mm path length. The spectra in the range of 350 nm to 190 nm were collected and corrected for buffer background.

### Cross-linking mass spectroscopy

The protein was centrifuged at 17,000*g*, and the supernatant was collected and re-quantified. One hundred μg tau was used for the cross-linking reaction in 1x PBS buffer, pH 7.4, with 1 mM disuccinimidyl suberate (DSS) (Thermo Fisher Scientific) and incubated at RT with 350 rpm shaking for 1 min. The reaction was quenched by 100 mM NH_4_HCO_3_, pH 7.8, at 37 °C for 30 min and evaporated to dryness. Samples were resuspended in 8 M urea, reduced with 10 mM DTT at 37 °C for 30 min, and alkylated with 5 mM iodoacetamide at RT for 30 min. The samples were then diluted to 1 M urea with 50 mM NH_4_HCO_3_ buffer, pH 7.8. Trypsin digestion was carried out at an enzyme-to-protein ratio of 1:50 at 37 °C overnight. After acidification with formic acid to 2% (v/v), the samples were desalted by Pierce C18 Tips (Thermo) and evaporated to dryness. Four replicates of wild-type and ΔK280 studies were carried out. Mass spectrometry data was filtered by a score of 25. Links of lysine-lysine pairs present in all replicates were defined as consensus cross-links. Intramolecular cross-links were visualized by xiNET (34). The average frequency of modification was converted into a heat map and the number of cross-links connected each domain to the R2 or R3 domain was counted.

### Denaturation

Tau at 1 μM was incubated in 20 mM Tris buffer, pH 7.4, containing different concentrations of GdnHCl ranging from 0 to 3 M at 25 ^o^C overnight to reach equilibrium. Compound 4, 4′-dianilino-1, 1′-binaphthyl-5, 5′-disulfonic acid (Bis-ANS) was added to a final concentration at 2 μM and the fluorescence intensity was measured with excitation at 400 nm and emission at 470 nm to 530 nm at 25 ^o^C. Each data point was run at least in triplicate. The data were averaged and fitted with a two-state folding (N↔U) model with the following equation.

$$y_{fit}=\frac{y_{N}+m_{N}\left[D\right]+\left(y_{U}+m_{U}\left[D\right]\right)e^{-\left(\Delta G_{UN}^{H_{2}O}+m\left[D\right]\right)/RT}}{1+e^{-\left(\Delta G_{UN}^{H_{2}O}+m\left[D\right]\right)/RT}}$$

where, $y_{N}$ is the y-intercept and $m_{N}$ is the slope of linear regression of the pre-transition state, $y_{U}$ is the y-intercept and $m_{U}$ is the slope of linear regression of the post-transition state, [D] is the concentration of GdnHCl, $\Delta G^{o}$ is the free energy change in the absence of denaturant and m is the cooperativity index, R is the gas constant and T is the temperature in Kelvin.

### Analytical ultracentrifugation

Freshly prepared tau variants at 5 μM were examined in a ProteomeLab XL-I centrifuge with an An-60Ti rotor (Beckman Coulter) and SV experiment of AUC was performed (16). Briefly, 400 μl of 5 μM tau variants and 450 μl of buffer were loaded in 12-mm aluminum double-sector centerpieces and centrifuged at 50,000 rpm for 15 h at 25 °C. Ultraviolet absorption was adopted with the time interval of 2 min between each scanning. Moving boundaries were analyzed by SEDFIT software from the National Institutes of Health (NIH) and parameters were calculated by SEDNTERP software developed by the University of New Hampshire.

### Aggregation

Five μM tau in 20 mM Tris buffer, pH 7.4 was incubated with the presence of 10 μM ThT and heparin in a tau-to-heparin molar ratio of 4:1. A 40 μl aliquot of solution was placed in each well of a 384-well plate, and the fluorescence intensity was measured by SpectraMax M5 microplate reader (excitation: 442 nm; emission: 485 nm). Each sample was performed in triplicate.

### Seeding assay

Five μM WT and ΔK280 Tau monomers were incubated in 20 mM Tris buffer with or without the presence of a 5% v/v ratio of WT and ΔK280 preformed seeds, respectively, 10 μM ThT and heparin in a ratio of tau-to-heparin of 4:1, under 30 s agitation time. A 40 μl aliquot of the solution was placed in a 384-well plate, and the fluorescence intensity was measured using SpectraMax M5 microplate reader (excitation: 442 nm; emission: 485 nm). Each experiment was repeated three times.

### Transmission electron microscopy

The final product (10 μl) from the aggregation and seeding assay was applied onto 400-mesh Formvar carbon-coated copper grids (EMS Inc., Hatfield) for 5 min, rinsed, and then negatively stained by 2% uranyl acetate. The samples were examined with a Hitachi H-7000 transmission electron microscope (TEM) (Hitachi Inc.) with an accelerating voltage of 120 kV.

### Fourier-transform infrared microscopy

Conformation of the final product from aggregation and seeding assay was further analyzed with ATR-Fourier-transform infrared microscopy (FTIR). Fibrils were first centrifuged at 17,000*g* for 30 min at 4 ^o^C and washed with ddH_2_O twice, then resuspended in D_2_O before being applied onto PIKE Miracle ATR sampling accessory and dried with cool air. Data between 1,600 and 1,700 cm^−1^ were collected and normalized.

### Preparation of tau fibril seeds

WT or mutant tau at 5 μM was incubated with 1.25 μM heparin for 4 d to form fibrils. The preformed tau fibrils were sonicated using Ultrasonic Cleaner 3510 (Branson) for 20 min before the experiments.

### Fluorescence cross-correlation spectrometry (FCCS)

HEK293T cells were seeded at 20% confluence and transfected for tau overexpression by Lipofectamine 2000 (Thermo Fisher Scientific). The following FCCS measurements were conducted on a PicoQuant-TCSPC-module-equipped Leica TCS SP5 SMD confocal microscope. By using a × 63 water immersion objective with Argon 488 nm and He-Ne 594 nm laser pulse sources and Single Photon Avalanche Diodes (SPAD) photon counting detectors, cross-correlations of GFP-tagged and mCherry-tagged tau in wild-type and mutant forms in live cells were excited for 120 s. Diffusion coefficients in every sample were analyzed by QuickFit 3.0 3D normal diffusion model (DKFZ). The fitting function was described previously (35). Particle mass was calculated from the diffusion coefficient by the Stokes-Einstein equation. The particle size of each tau construct in the cell was calibrated by a control cell expressing eGFP-mCherry fusion protein.

### Flow cytometry and cell-to-cell transmission

HEK293T cells were transfected with the tau constructs. Two cells of interest were co-cultured in equal amounts to reach 80% confluence in 6-well plates on the second day. After 48 h, the cells were harvested and resuspended in PBS in the presence of 2% paraformaldehyde. A total of 15,000 cells were analyzed in each experimental condition using a FACS Aria II cytometer (BD Biosciences) equipped with 488- and 561-nm lasers with FITC and yellow PE-Texas red filters. Results from each experiment were quantified by FlowJo software (FlowJo LLC), with each experiment performed in triplicate. The transmission was indicated by the percentage of cells with dual fluorescence in all fluorescent cells (*i.e.*, single and double fluorescence).

For the transmission study with tau fibril and monomer seeds, HEK293T cells were transfected with the tau construct using Lipofectamine 2000. After 24 h, cells expressing eGFP-tau and mCherry-tau were co-cultured at 6 × 10^5^ cells each/well in 6-well plates. After co-culture, the cells were treated with buffer containing 20 mM Tris-HCl, pH 7.4, or fibril or monomer seeds at 5 μM from WT tau or mutant tau with Lipofectamine 2000, respectively. After four or 24 h incubation, the cells were harvested and fixed with 2% paraformaldehyde. Flow cytometry was used to quantify dual positive cells by analyzing 1 × 10^4^ cells in each sample. The eGFP-tau and mCherry-tau transfected cells cultured separately were mixed immediately before flow cytometry as a negative control. Each experiment was performed in triplicate. The transmission percentage was calculated in the second quadrant where mCherry and GFP signals co-exist in the same cell and normalized with the total signal in all four quadrants. This averaged out the possible difference in expression level. Statistical analysis was performed using GraphPad Prism (GraphPad Software) in two-way ANOVA with Tukey’s test (∗, *p* < 0.05; ∗∗, *p* < 0.01; ∗∗∗, *p* < 0.001).

## Data availability

Data can be provided upon reasonable request.

## Supporting information

This article contains supporting information.

## Conflict of interest

The authors declare that they have no conflicts of interest with the contents of this article.

## Supporting information

Supplemetary Figures
