# Supplementary material for: Tau destabilization in a familial deletion mutant K280 accelerates its fibrillization and enhances the seeding effect
Source: J Biol Chem. 2025 Jan 13;301(2):108184. doi: 10.1016/j.jbc.2025.108184 (PMC11849630; doi:10.1016/j.jbc.2025.108184)
Supplement: Supplemetary Figures [file mmc1.pdf]

# Supplementary Information

## Tau destabilization in a familial deletion mutant K280 accelerates its fibrillization and modulates the seeding effect

Gary Jen-Wei Chen[1§], Ming-Yun Chang[1,2§], Xin-Peng Lin[1,3], Debapriya Kundu[1,4,5], Yu-Jen Chang[1,6], Yun-Ru Chen[1,2,3,4,6*]

## Supplementary Figures

### Supplementary Figure 1

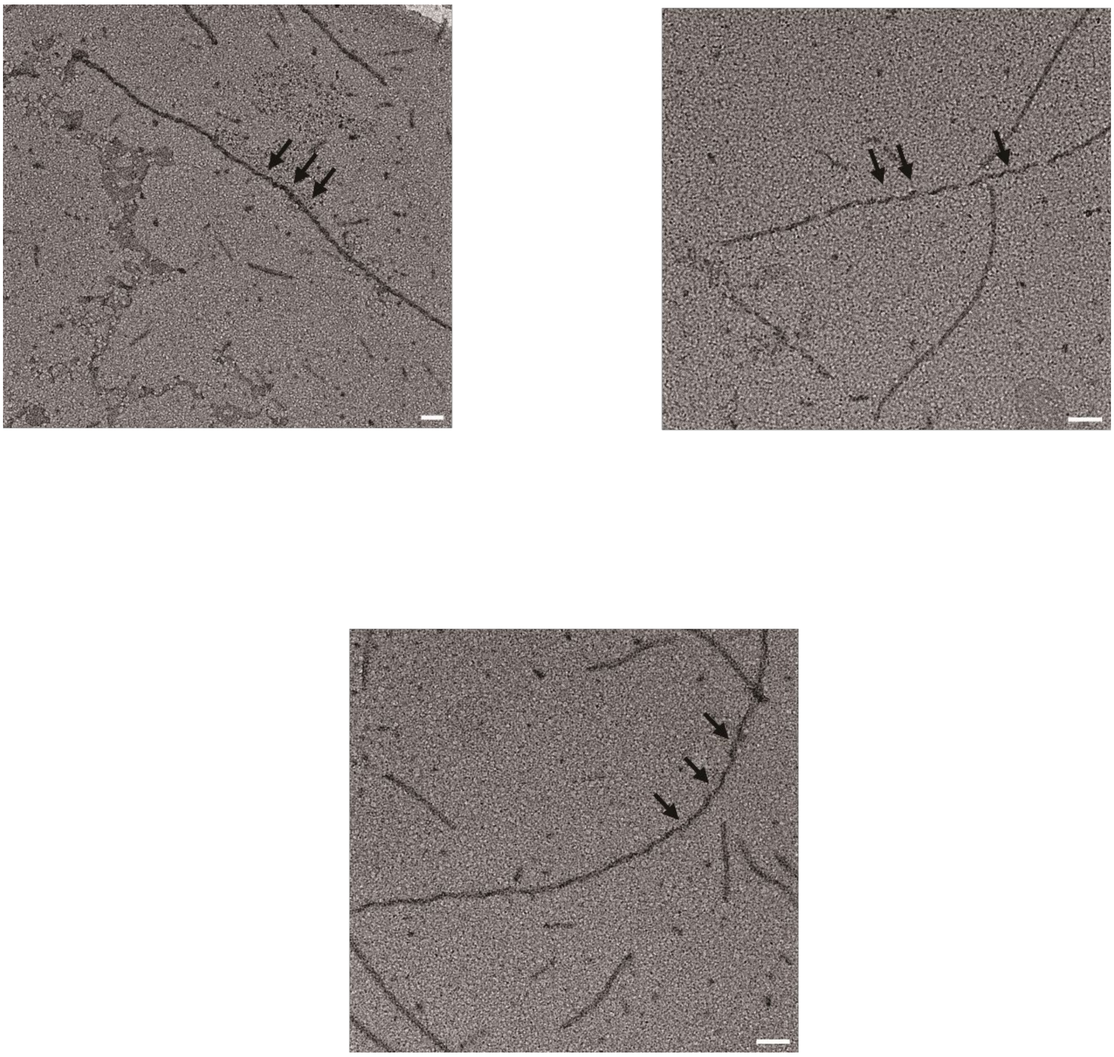

**Figure S1. TEM images of Tau ΔK280 mutant fibrils.** The fibrils were collected from the end-point of the fibrillation assay. The periodic twits are indicated by arrow heads (black). The scale bar is 100 nm. The SI figures were used for quantification of the samples including the representative image used in Figure 4D.

**Supplementary Figure 2.**

A

B

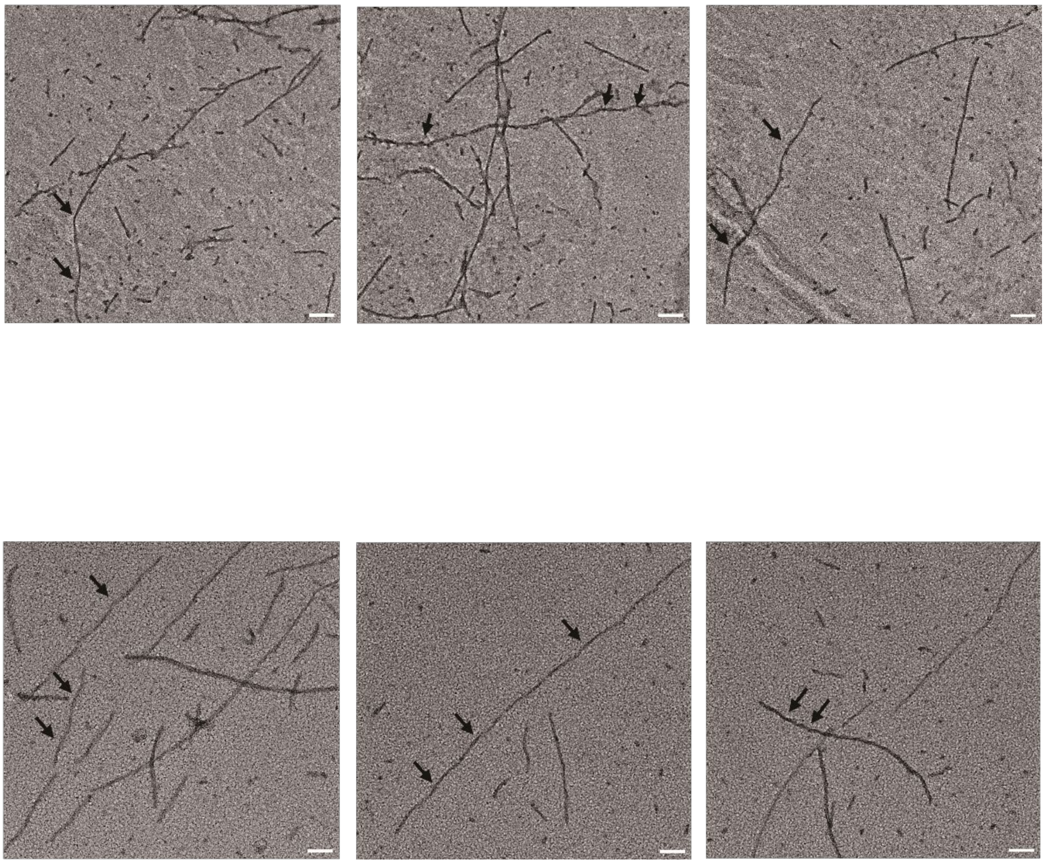

**Figure S2. TEM images of fibrils from the seeding assay.** The end-point product of tau ΔK280 mutant monomer seeded with wild type fibril seeds (A) and ΔK280 mutant fibril seeds (B) were imaged. The periodic twits are indicated by arrow heads (black). The scale bar is 100 nm. The SI figures were used for quantification of the samples including the representative image used in Figure 5.

**Supplementary Figure 3**

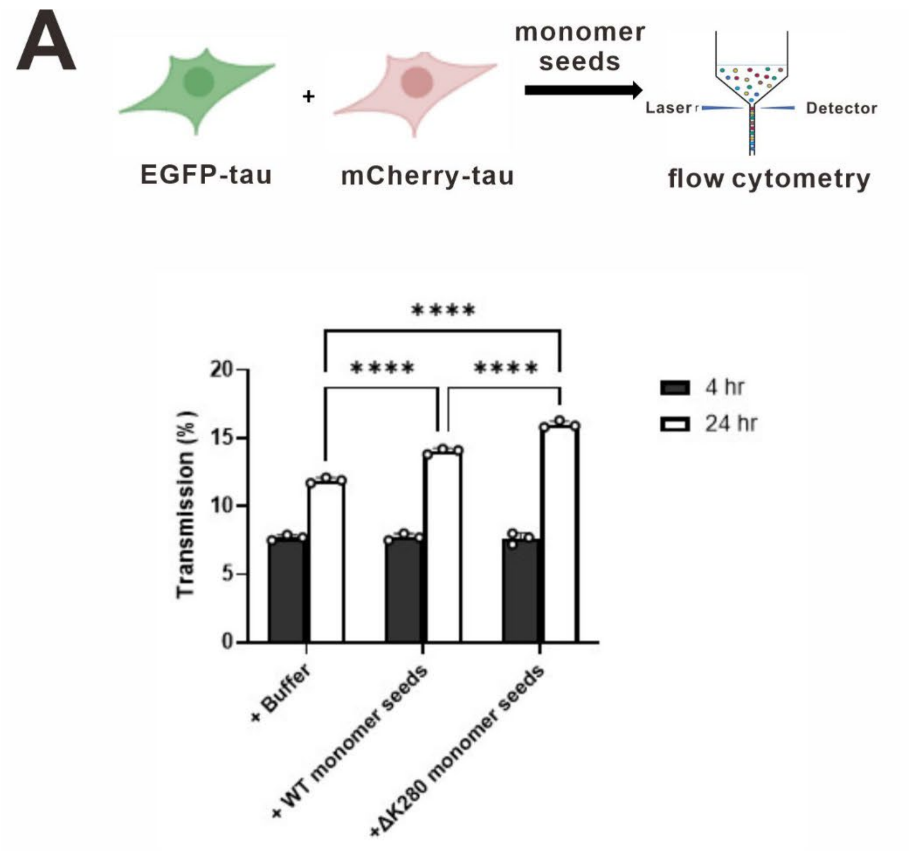


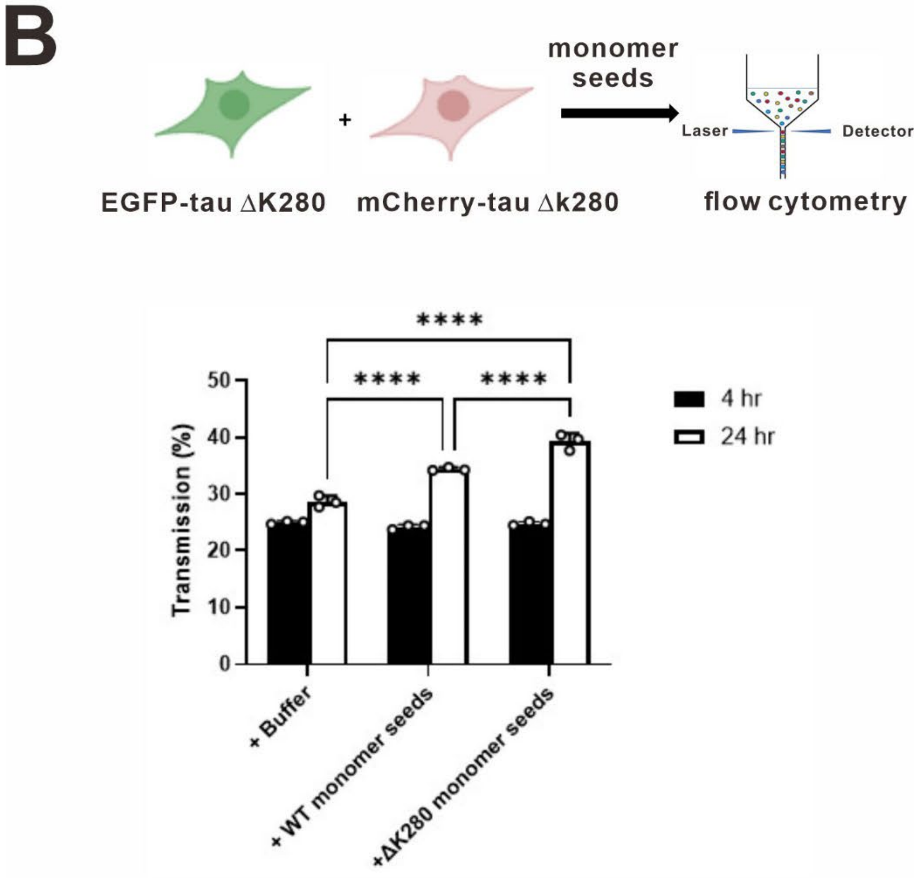

**Figure S3. Cell-to-Cell transmission of Tau with Tau monomer seeds by flow cytometry. (A)** Pictorial representation of eGFP-tau (green) and mCherry-tau (red) overexpressed in HEK293 cells, followed by co-culture in the presence of buffer or tau monomer seeds and subsequent flow cytometry at 4 and 24 h respectively. **(B)** Percentage of transmission in the co-cultured cells expressing eGFP-tau WT and mCherry-tau WT treated with WT or mutant tau monomer seeds for 4 and 24 h, respectively. **(C)** Percentage of transmission in the co-cultured cells expressing eGFP-tau ΔK280 and mCherry-tau ΔK280 treated with WT or mutant tau monomer seeds for 4 and 24 h, respectively. The statistical analysis was performed by two-way ANOVA with Tukey's multiple comparison post-test (*, $p<0.05$; **, $p<0.01$, ***, $p<0.001$, ***, $p<0.0001$).
